# Trajectories of squamous cell carcinoma antigen and outcomes of patients with advanced penile cancer after chemotherapy based on paclitaxel, ifosfamid, and cisplatin regimen

**DOI:** 10.1002/cam4.7353

**Published:** 2024-06-18

**Authors:** Nan Ma, Yi‐Xiang Gan, Yin‐Yao Chao, Zhen‐Hua Liu, Xian‐Da Chen, Kai Yao, Hui Han, Sheng‐Jie Guo

**Affiliations:** ^1^ State Key Laboratory of Oncology in South China, Collaborative Innovation Center for Cancer Medicine, Department of Urology Sun Yat‐sen University Cancer Center Guangzhou China; ^2^ State Key Laboratory of Oncology in South China, Guangdong Provincial Clinical Research Center for Cancer, Department of Liver Surgery Sun Yat‐sen University Cancer Center Guangzhou P.R. China; ^3^ Zhongshan School of Medicine, Sun Yat‐sen University Guangzhou P.R. China

**Keywords:** biomarker, penile cancer, squamous cell carcinoma antigen, survival, TIP chemotherapy, tumor response

## Abstract

**Introduction:**

Penile cancer (PC) is a lethal malignancy with no effective prognostic biomarker. We aim to investigate associations between trajectories of squamous cell carcinoma antigen (SCC‐A) and patient outcomes after chemotherapy based on paclitaxel, ifosfamid, and cisplatin (TIP) regimen.

**Methods:**

Consecutive AJCC staging III/IV PC patients who received TIP chemotherapy and repeated SCC‐A measurements in 2014–2022 were analyzed. Latent class growth mixed (LCGM) models were employed to characterize patients' serum SCC‐A trajectories. Patient survival, and clinical and pathological tumor responses were compared. Inverse probability treatment weighting was used to adjust confounding factors.

**Results:**

Eighty patients were included. LCGM models identified two distinct trajectories of SCC‐A: low‐stable (40%; *n* = 32) and high‐decline (60%; *n* = 48). Overall survival (HR [95% CI]: 3.60 [1.23–10.53], *p* = 0.019), progression‐free survival (HR [95% CI]: 11.33 [3.19–40.3], *p* < 0.001), objective response rate (37.5% vs. 62.5% *p* = 0.028), disease control rate (60.4% vs. 96.9% *p* < 0.00), and pathological complete response rate (21.2% vs. 51.9%, *p* = 0.014) were significantly worse in the high‐decline arm.

**Conclusion:**

PC patients' SCC‐A change rate was associated with tumor response and patient survival after TIP chemotherapy. SCC‐A might assist tumor monitoring after systemic therapies.

## Graphical abstract

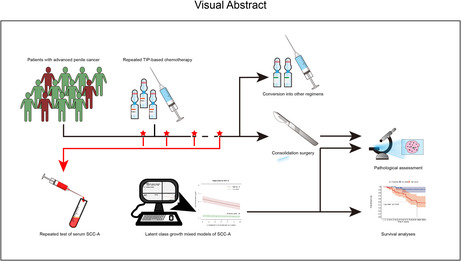

## 1 INTRODUCTION

Penile cancer (PC) is a rare yet highly lethal malignancy.^1^ In economically undeveloped regions of the world, it even accounts for nearly 10% of cancer burdens.^2^, ^3^ For advanced PC cases, a multimodal strategy which combines chemotherapy and consolidation surgery shows great advantages.^4^, ^5^ Paclitaxel, ifosfamide, and cisplatin (TIP) chemotherapy is a well‐known regimen which could induce marked tumor responses and thus better prognosis.^6^, ^7^, ^8^ Guidelines now recommend TIP regimen as a first‐line palliative therapy for advanced PC.^4^

Serum biomarkers is reliable, noninvasive, and cost‐effective tools which assist diagnosis, prognostic prediction and personalized treatment of cancer. Squamous cell carcinoma antigen (SCC‐A) is a tumor‐associated glycoprotein. Since penile squamous cell carcinomas (PSCCs) are the major histological type which originated from the squamous cells of the glanular and preputial skin, SCC‐A tends to be markedly elevated at diagnosis.^9^ Associations between serum SCC‐A levels and lymph node (LN) metastasis was observed.^10^, ^11^, ^12^ Several studies also indicated patients with higher SCC‐A levels had poorer survival after surgery.^11^, ^12^ However, for advanced cases, whether SCC‐A could reflect drug efficacy and assist disease monitoring remains unknown. In addition, the role of SCC‐Ag change, including change amplitude and rate, is hardly defined. New methods are necessary for assessment of utility of SCC‐A variation as a surrogate biomarker.

Recently, latent class growth mixed (LCGM) models have emerged as powerful tools to fit dynamic changes of parameters.^13^, ^14^, ^15^ Unlike conventional studies which focused on biomarker measured at single or limited timepoints, LCGM models reflected longitudinal variations of biomarkers and revealed their significance in long‐term monitoring of cancer.

In this observational study, we aim to investigate whether serum SCC‐A trajectories are associated with PC patient outcomes after TIP‐based chemotherapy.

## 2 MATERIALS AND METHODS

### 2.1 Study design and patient inclusion

This retrospective observational study has gained approval (approval number: B2023‐390‐01; approval date: August 16, 2023) from the ethics committee of the Sun Yat‐sen University Cancer Center (SYSUCC). The informed consent requirement was waived and patient information was kept confidential. Ethical guidelines of Declaration of Helsinki were complied with. We reported this work adhering to REMAKR reporting criteria.^16^

**FIGURE 1:**
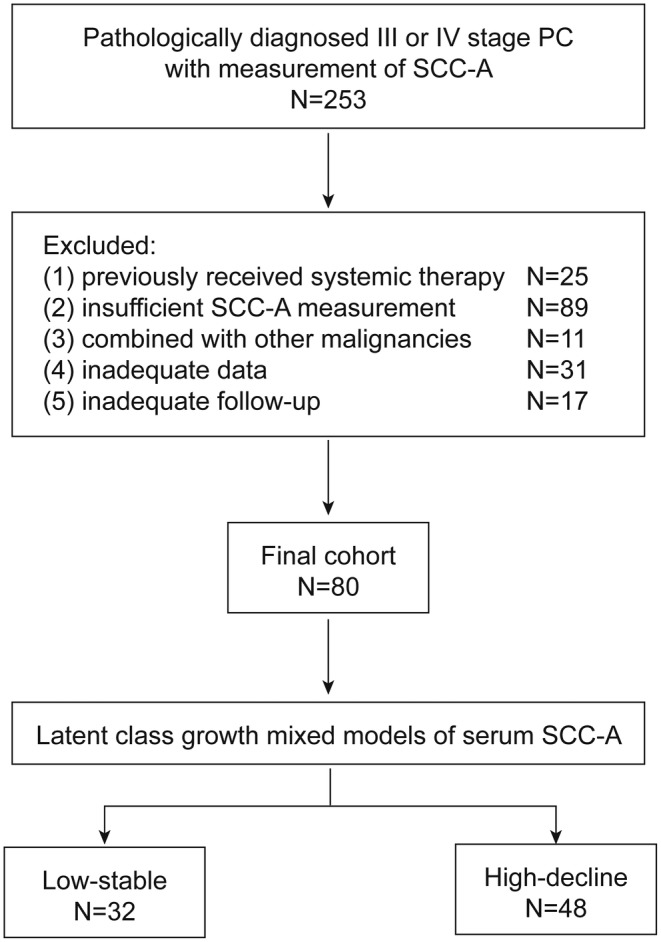
Patient inclusion and study design.

Advanced PC patients who received TIP chemotherapy at SYSUCC from January 1, 2014 to December 31, 2022 were candidate study subjects (Figure 1). The inclusion criteria were as follows: (1) pathologically diagnosed with PC; (2) had LN or distant metastasis; (3) received TIP chemotherapy between January 1, 2016 and December 31, 2022; and (4) received at least one pre‐chemotherapy and two repeated post‐chemotherapy measurements of SCC‐A. The exclusion criteria were as follows: (1) previously received other systemic therapies; (2) combined with other squamous cell derived malignancy; (3) inadequate data; and (4) follow‐up less than 6 months. Pre‐chemotherapy serum SCC‐A levels was defined as their measurements within 1 month before TIP chemotherapy. Post‐chemotherapy serum SCC‐A levels included the values before any other therapy at each follow‐up record after TIP chemotherapy. Patients' demographic information, treatment details, laboratory testing results, imaging examination results, and pathological outcomes were collected from the electronic medical record system of SYSUCC.

### 2.2 Treatment process

Throughout the treatment, decisions on systemic therapies and consolidation surgeries were made by a multidisciplinary team which was comprised of urologists, oncologists, radiologists, and anesthetists. Protocols of TIP regimen as well as the combination with targeted and immune therapies were previously described.^17^ Specifically, after diagnosis of advanced PC (AJCC staging III or IV), patients received the TIP chemotherapy regimen (albumin‐bound paclitaxel 260 mg/m^2^, Day 1; cisplatin 25 mg/m^2^, Days 1–3, ifosfamide 1200 mg/m^2^, Days 1–3) intravenously every 3 weeks, with no more than 5 cycles, or until dose‐limiting toxicity or disease progression or patient withdrawal. For those with combined use of targeted and immune therapies, nimotuzumab (anti‐EGFR monoclonal antibody, Biotech Pharmaceutical Co., Ltd, Beijing, China) 400 mg and toripalimab (anti‐PD‐1 monoclonal antibody, Junshi Biosciences Co., Ltd., Shanghai, China) 240 mg were injected intravenously on Day 1. Consolidation surgery was assessed and performed after systemic therapy. Surgical criteria were as follows: (1) disease reached complete response (CR), partial response (PR), or stable disease (SD) according to the Response Evaluation Criteria in Solid Tumors (RECIST, version 1.1)^18^; (2) absence of contraindications of general surgery. Surgical type included penectomy (partial or total), modified radical inguinal lymph node dissection (unilateral or bilateral, ILND), pelvic lymph node dissection (unilateral or bilateral, PLND), and adjacent flap transfer, based on preoperative radiographic examination. Surgeries were performed by at least one senior urologist.

### 2.3 Measurement of SCC‐A

Measurements of SCC‐A were conducted using blood samples at clinical laboratory of SYSUCC. Serum SCC‐A was measured by electrochemiluminescence immunoassay (Roche, Shanghai, China). The upper limits of the normal reference values of SCC‐A were 1.5 ng/mL.

### 2.4 Classification of longitudinal SCC‐A data using LCGM models

LCGM models were employed to identify patients with distinct longitudinal trajectories of post‐chemotherapy serum SCC‐A levels. Serum SCC‐A values were log‐transformed by natural logarithm (ln transformation) due to their left skewness. Estimation of extended mixed models using latent groups and processes were performed using the package lcmm (version 2.0.0, https://cecileproust‐lima.github.io/lcmm/) for R (Version 4.1.1). We set ln[SCC‐A] as linear, quadratic or cubic function of time with 1–3 potential classes with the same starting values derived from the 1‐class model. Criteria for the optimal‐fit model were as follows: (1) significant improvement of the model in Bayesian information criterion (BIC); (2) high mean posterior probabilities (>0.80); (3) no less than 10% participants in any single trajectory class; and (4) the latent classes fit realistic clinical meaning.

### 2.5 Assessment of tumor response

Patients' clinical responses were assessed in according with the RECIST 1.1 through enhanced computed tomography (CT) or positron emission tomography/computed tomography (PET‐CT). For those who underwent penectomy and/or local lymphadenectomy, pathological analyses were performed via hematoxylin–eosin staining (H&E) staining and immunohistology staining. Pathological complete response (PCR) was defined with no viable tumor cells in the penile and in all the local lymph nodes after TIP chemotherapy.

### 2.6 Follow‐up and study endpoint

The primary outcome was overall survival (OS) and progression‐free survival (PFS). OS is defined by the duration between date of first cycle of chemotherapy and date of death or lost follow‐up. PFS is defined by the duration between date of first cycle of chemotherapy and date of observation of PD, death, or lost follow‐up. Patients were followed up by outpatient service or telephone contact. Generally, PC patients discharged after surgery were followed up once every 1–3 months for the initial half year, and every 3–6 months thereafter. Surveillance instruments were CT scans and/or PET‐CT, as well as laboratory tests including blood routines, liver function, renal function, coagulation function, and urinary tumor markers. Follow‐up data were recorded by electronic medical record system of SYSUCC. The date of the final follow‐up was December 31, 2023. Secondary outcomes were objective response rate (ORR), disease control rate (DCR), and PCR. ORR is defined by the rate of complete or partial response, while DCR is defined by the rate of complete response, partial response, and stable disease according to RECIST 1.1.^18^

### 2.7 Statistics

Continuous variables were compared using the Wilcoxon rank sum test. Categorical variables were compared using Pearson's chi‐square test or Fisher's exact test, as appropriate. Kaplan–Meier methods were used to visualize associations of SCC‐A trajectories with OS. To balance confounding factors, we employed inverse probability treatment weighting (ITPW) with adjustment for age (>60 or ≤ 60 years old), Eastern Cooperative Oncology Group (ECOG) performance status (0, 1 or 2), smoking (yes or no), AJCC stage, combined targeted and immune therapies (yes or no, inhibitors of epidermal growth factor receptor [EGFR] and inhibitor of programmed cell death protein 1 [PD‐1] or its ligand [PD‐L1]), consolidation surgery (yes or no), tumor differentiation (poor, moderate to well), as well as preoperative SCC‐A levels. Propensity scores (PS) were calculated based on logistic regression models. Patients with different SCC‐A trajectories were weighted with inverse PS and inverse of 1 minus the PS, respectively. A two‐tailed *p* value was considered to be statistically significant. All data processing and statistical analyses were performed and visualized using R statistical software (Version 4.1.1, https://www.r‐project.org).

## 3 RESULTS

### 3.1 Patient characteristics

Of 231 patients who received TIP chemotherapy and measurements of SCC‐Ag, 80 were finally included in this study. Patient characteristics are summarized in Table 1. Before treatment, 66 (82.5%) patients had elevated serum SCC‐A levels. Twenty‐five (31.3%) and 55 (68.8%) patients were diagnosed as AJCC III and IV PC, respectively. Fifty‐nine (73.8%) patients received previous non‐systemic therapies. Twelve patients (15%) had distant metastasis. Median cycle of TIP chemotherapy was four. Sixty (75%) patients received combined use of targeted and immune therapies.

**TABLE 1:** Patients' characteristics.

Characteristics	High‐decline (*N* = 48)	Low‐stable (*N* = 32)	Overall (*N* = 80)	*p* value
Age, years	57 (49–66)	56 (50–66)	56 (49–66)	0.953
Smoking history	21 (43.8%)	10 (31.3%)	31 (38.8%)	0.236
ECOG PS				
0	9 (18.8%)	8 (25.0%)	17 (21.2%)	
1	24 (50.0%)	23 (71.9%)	47 (58.8%)	
2	15 (31.6%)	1 (3.1%)	16 (20.0%)	
BMI, kg/m^2^, median	21.7 (20.3–23.3)	24.2 (21.9–27.3)	22.9	0.008
TNM staging				
III	9 (18.8%)	16 (50.0%)	25 (31.3%)	0.003
IV	39 (81.3%)	16 (50.0%)	55 (68.8%)	
Tumor differentiation				
Poor	23 (47.9%)	16 (50.0%)	39 (48.8%)	0.855
Moderate to well	25 (52.1%)	16 (50.0%)	41 (51.2%)	
Distant metastatic lesions				
None	38 (79.2%)	29 (90.6%)	67 (83.8%)	0.174
Bone	4 (8.3%)	1 (3.1%)	5 (6.3%)	0.637
Lung	2 (4.2%)	1 (3.1%)	2 (2.5%)	1.000
Back muscle	4 (8.3%)	0	4 (5.0%)	0.249
Abdominal lymph node	0	1 (3.1%)	1 (1.3%)	0.400
Baseline SCC‐A level, ng/mL	11.8 (7.4–34.8)	1.8 (1.2–2.6)	6.6 (1.9–15.6)	<0.001
Chemotherapy, no. (%)				
TIP	12 (25.0%)	8 (25.0%)	20 (25.0%)	1.000
TIP+Anti‐PD1 + anti‐EGFR	36 (75.0%)	24 (75.0%)	60 (75.0%)	
Treatment cycles	4 (3–4)	4 (3–4)	4 (3–4)	0.444
Prior treatment, no. (%)				
None	11 (22.9%)	10 (31.3%)	21 (26.3%)	0.407
Partial penectomy	27 (56.3%)	18 (56.3%)	45 (56.3%)	1.000
Radical penectomy	9 (18.8%)	4 (12.5%)	13 (16.3%)	0.458
ILND	18 (37.5%)	6 (18.8%)	24 (30.0%)	0.073
PLND	3 (6,3%)	0	3 (3.8%)	0.400
Radiotherapy	4 (8.3%)	1 (3.1%)	5 (6.3%)	0.637
Other	1 (2.1%)	0	1 (1.3%)	1.000

*Note*: Continuous variables were presented as median (inter‐quartile range). Categorical variables were presented as number (%).

Abbreviations: BMI, body mass index; ECOG PS, Eastern Cooperative Oncology Group performance status; EGFR, epidermal growth factor receptor; ILND, inguinal lymph node dissection; PD1, programmed cell death protein 1; PLND, pelvic lymph node dissection.

### 3.2 Identification of trajectories of SCC‐Ag

**FIGURE 2:**
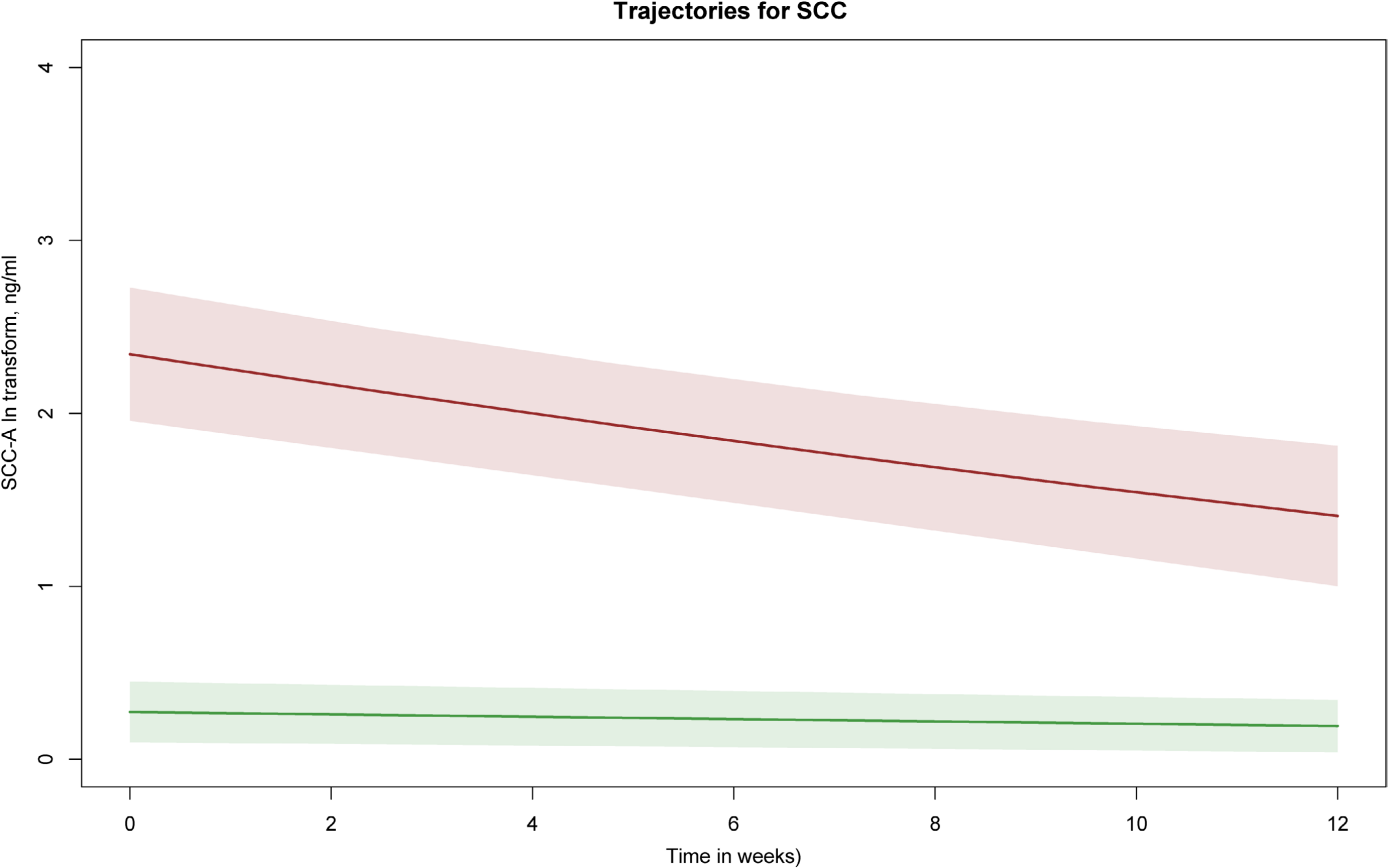
Visualization of latent class mixed models.

Fitting process for 1 through 3 classes by LCGM models is summarized in Table 2. Finally, a model of quadratic parameters with two classes was optimal according to the selection criteria. Predicted mean trajectories of SCC‐A are depicted in Figure 2. Based on their levels and tendencies, the two distinct trajectories were labeled as: low‐stable (40%; *n* = 32) and high‐decline (60%; *n* = 48). The mean SCC‐A levels maintained below the upper reference limit of normal range (1.5 ng/mL) across the treatment process. There is no overlap between the 95% confidence interval (95% CI) of the two identified trajectories. Patient characteristics are summarized in Table 1. More patients were diagnosed as AJCC IV PC (*n* = 39, 81.3%) in the high‐decline class. Before chemotherapy, median serum SCC‐A levels were significantly higher in the high‐decline class than the low‐stable class (11.8 vs. 1.8, P<0.001).

**TABLE 2:** Latent class growth mixture model (LCGMM) results of SCC‐A model fitting process.

No. of latent classes	Polynomial degree	BIC	Log‐Lik	% Participants per class	Mean posterior probabilities	% Posterior probabilities >70%
1	Linear	929.1	−451.4	100	NA	NA
	Quadratic	929.6	−449.4	100	NA	NA
	Cubic	932.6	−448.8	100	NA	NA
2	Linear	904.8	−430.5	43.8/56.2	0.921/ 0.960	97.1/97.8
	Quadratic	893.9	−420.7	40.0/60.0	0.854/0.973	78.1/97.9
	Cubic	2e^9	‐e^9	NA	NA	NA
3	Linear	909.1	−423.9	48.8/22.5/28.7	0.941/0.827/0.937	94.9/72.2/95.7
	Quadratic	913.1	201.7	41.3/38.7/20.0	0.827/ 0.837/0.896	78.8/77.4/81.3
	Cubic	2e^9	‐e^9	NA	NA	NA

### 3.3 Survival analyses

**FIGURE 3:**
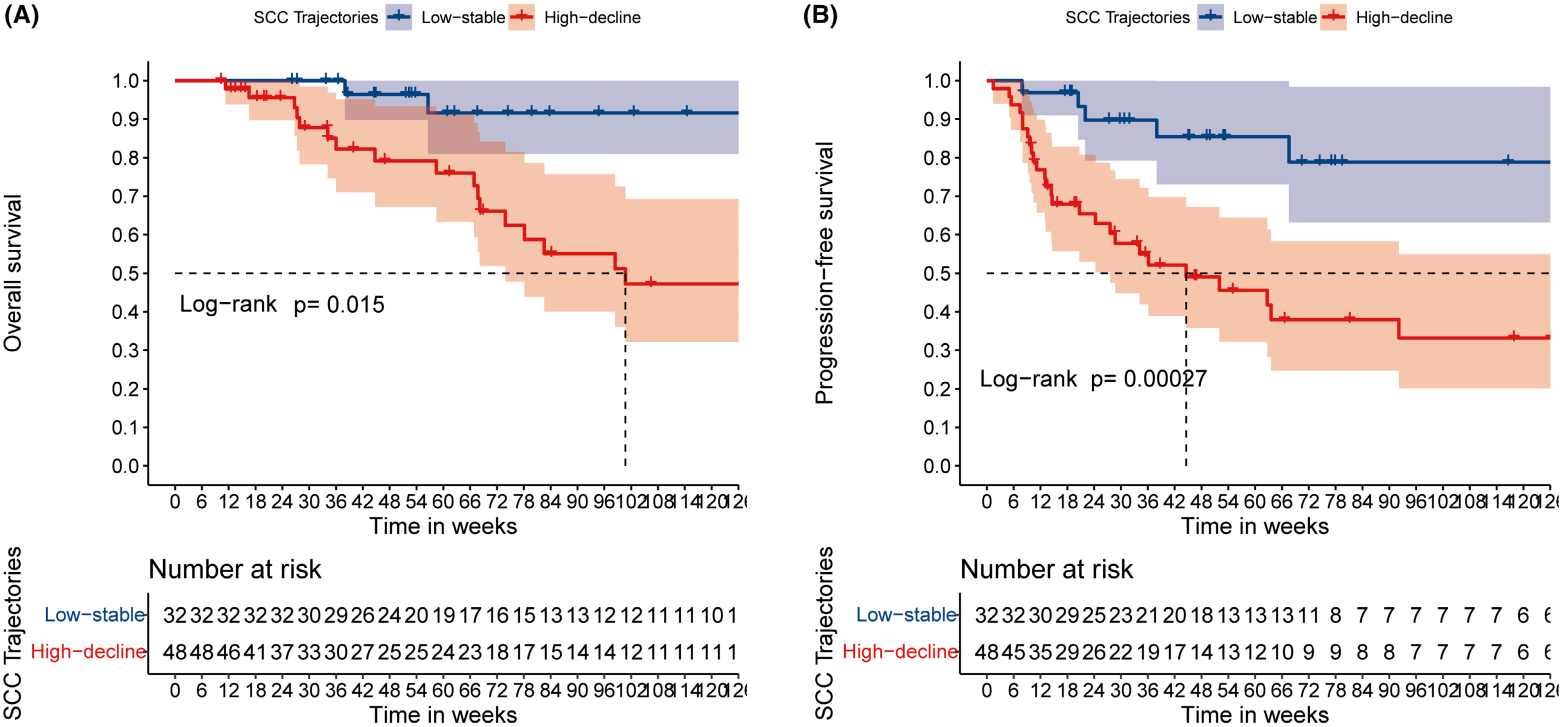
K–M curves of OS (A) and PFS (B) of patients with different SCC‐A trajectories.

Median follow‐ups were 59.8 and 64.5 weeks in the high‐decline class and the low‐stable class of SCC‐A trajectories, respectively. Patients of the low‐stable class showed better OS than those of the high‐decline class, with median OS of unreached versus 101 (95% CI: 73.9‐NA) weeks (*p* = 0.015, Figure 3A). The low‐stable class also showed better PFS than those of the high‐decline class, with median PFS of unreached versus 44.6 (95% CI: 27.6‐NA) weeks (*p* < 0.001, Figure 3B). After ITPW adjustment with age, AJCC staging, ECOG status, combined targeted and immune therapies, consolidation surgery, tumor differentiation, as well as baseline SCC‐A levels, patients of the high‐decline class still showed a higher risk of death (HR [95% CI]: 3.60 [1.23, 10.53], *p* = 0.019; Table 3) and disease progression (HR [95% CI]: 11.33 [3.19, 40.3], *p*<0.001; Table 3). Conversely, high levels of baseline serum SCC‐A (>1.5 ng/mL) did not show better OS (HR [95% CI]: 1.34 [0.45, 3.99], *p* = 0.594) or PFS (HR [95% CI]: 1.32 [0.51, 3.43], *p* = 0.569) than the low group (≤1.5 ng/mL).

**TABLE 3:** ITPW adjusted COX regression analyses of patients' survival by baseline SCC‐A or SCC‐A trajectories.

Class	*N*	Non‐adjusted	*p* value	Adjusted^b^	*p* value I	Adjust II^a^	*p* value II
OS							
Baseline SCC‐A	80						
Low (≤1.5 ng/mL)	14	Reference		Reference			
High (>1.5 ng/mL)	66	1.34 (0.45, 3.99)	0.594	0.61 (0.12, 3.01)	0.550		
SCC‐A trajectories							
Low‐stable	32	Reference		Reference		Reference	
High‐decline	48	3.21 (1.19, 8.69)	0.021	3.07 (1.05, 8.95)	0.040	3.60 (1.23, 10.53)	0.019
PFS							
Baseline SCC‐A	80						
Low (≤1.5 ng/mL)	14	Reference		Reference			
High (>1.5 ng/mL)	66	1.32 (0.51, 3.43)	0.569	1.62 (0.63, 4.16)	0.314		
SCC‐A trajectories							
Low‐stable	32	Reference		Reference		Reference	
High‐decline	48	4.98 (1.91, 12.96)	0.001	5.86 (2.03, 16.93)	0.001	11.33 (3.19, 40.3)	<0.001

Abbreviations: OS, overall survival; PFS, progression‐free survival.

^a^ Further adjusted by baseline SCC‐A level (≤1.5, >1.5 ng/mL).

^b^ Adjusted by age (≤60, >60 years old), AJCC staging (III or IV), ECOG status (0, 1, or 2), combined targeted and immune therapies (yes or no), consolidation surgery (yes or no), tumor differentiation (poor, moderate to well).

### 3.4 Clinical and pathological responses

Of the 48 patients in the high‐decline class, 33 (68.8%) patients underwent post‐chemotherapy surgery. Of the 32 patients in the low‐stable class, 27 (84.3%) patients underwent post‐chemotherapy surgery. Patients' clinical and pathological responses after TIP chemotherapy are summarized in Table 4. ORR in the high‐decline class and the low‐stable class of SCC‐A trajectories were 37.5% and 62.5%, respectively (*p* = 0.028). DCR in the high‐decline class and the low‐stable class of SCC‐A trajectories were 60.4% and 96.9%, respectively (*p* < 0.001). PCR in the high‐decline class and the low‐stable class of SCC‐A trajectories were 21.2% and 51.9%, respectively (*p* = 0.014).

**TABLE 4:** Clinical and pathological tumor responses.

	High‐decline (*N* = 48)	Low‐stable (*N* = 32)	*p* value
Clinical response			
Partial response.	18 (37.5%)	20 (62.5%)	
Stable response	11 (22.9%)	11 (34.3%)	
Progressive disease	19 (39.6%)	1 (3.1%)	
Objective response rate	18 (37.5%)	20 (62.5%)	0.028
Disease control rate	29 (60.4%)	31 (96.9%)	<0.001
Pathological response			
Pathologic complete response	7 (21.2%)	14 (51.9%)	0.013
Not evaluated	15 (31.3%)	5 (15.6%)	

*Note*: Categorical factors are presented as *n* (%).

## 4 DISCUSSION

In this study, we used longitudinal data to identify two distinct patterns of SCC‐A change in PC patients after TIP regimen and observed significant association between SCC‐A trajectories and patients' long‐term survival. Intriguingly, SCC‐A change rate, rather than baseline SCC‐A levels, was an independent prognostic factor. To the best of our knowledge, this is the first study which identified SCC‐A and its temporal changes as prognostic biomarkers in advanced, chemotherapy‐treated PC cases.

Because of the rare incidence and variable clinical manifestations, diagnosis of PC was usually delayed and many patients already had advanced disease at first diagnosis. For PC cases with the presence of LN metastasis, reported 5‐year survival rate ranged from 19.4% to 67.6%.^19^, ^20^, ^21^ TIP regimen has become the first‐line palliative therapy for advanced PCs.^4^ Nevertheless, so far, no reliable biomarker has been identified to assist diagnosis and prognostic prediction in mainstream practice. Candidate markers include SCC‐A, C‐reactive protein (CRP), neutrophil‐to‐lymphocyte ratio, Ki‐67, human papillomavirus (HPV), P16INK4a, TP53, and programmed death‐ligand 1 (PD‐L1).^9^, ^22^, ^23^, ^24^, ^25^ Among those, SCC‐A is a sensitive marker and feasible to be tested though blood sample. Two previous studies observed preoperative SCC‐A levels might be associated with patient survival. However, the association didn't reach statistical significance in multivariate COX regression models. In addition, these studies were both conducted in surgical cases. For assessment of efficacy of systemic therapies, due to the non‐curative nature, a static measurement of biomarkers tended to be insufficient to reflect sensitivity of cancer cells to drugs. The features of SCC‐A change, including change amplitude and rate may help monitor tumor responses and thus predict patient survival.

The LCGM model is an algorithmic tool which could integrate individual‐level longitudinal data and were widely used in public health studies.^13^, ^14^, ^15^ Recently, this novel methodology was also applied in biomarker investigations of other malignancies.^26^, ^27^, ^28^ In this study, we successfully identified two distinct patterns of SCC‐A change after TIP chemotherapy. We found patients with low‐stable trajectories had better survival than those with high‐decline trajectories. Until the date of last follow‐up, the low‐stable arm didn't reach median OS or PFS. After adjustment of confounders by ITPW, risks of death and progression of the high‐decline arm were still significantly higher than the low‐stable arm. To our knowledge, this is the first study in which SCC‐A was identified as an independent factor associated with patient survival in a multivariate model. Conversely, high levels of baseline serum SCC‐A were not significantly associated with survival (median [95% CI]: OS, 1.34 [0.45–3.99], PFS, 1.32 [0.51–3.43]).

Finally, we validated association between SCC‐A trajectories and patients' outcomes from the perspective of tumor's clinical and pathological responses. Surgical rate was lower in the high‐decline arm, majorly because of missing surgical indications for tumor progression. Patients with low‐stable trajectories of SCC‐A showed significant better ORR, DCR, and PCR than those with high‐decline trajectories. Intriguingly, of 48 patients in the high‐decline class, 33 (68.8%) patients underwent post‐chemotherapy surgery, lower than 27 (84.3%) patients in the low‐stable class. Actually, more patients in the high‐decline class suffered from swiftly progressive cancer and failed to tolerate surgical operations due to bad body performance. In some way, the different post‐chemotherapy surgical rates of the two groups reflects distinct chemotherapy efficacy. And multi‐variate adjustment has included whether receiving post‐chemotherapy surgery as a potential confounder. However, although the low‐stable group harbored relatively better tumor responses and survival, the therapeutic efficacy of the high‐decline group was still good. We considered it was greatly attributed to the success of introduction of targeted therapies and ICIs, as reported by recent studies.^17^, ^29^, ^30^ In our study, 75.0% of subjects in both arms underwent the triple medication. However, selection bias was inevitable for data requirement of construction of LCGM models. For those who suffered rapid progression of tumors, treatment schedules were more likely to be early modified. In other words, these patients failed to meet the inclusion criteria and treatment efficacy of the whole patients were overestimated. Given these, we are looking forward to prospective, large‐scale studies to validate the efficacy of the regimen.

Despite the novel findings, the study is still affected by some limitations. The major limitation is the limited sample size because of the low incidence of PC and the single‐center, retrospective nature of the study. Since there lacks high‐grade evidence supporting the prognostic significance of SCC‐A in systemic therapies of PC, only 80 out of the overall cohort of 231 patients received repeated SCC‐A measurement in clinical practice. Besides, the follow‐up time is relatively limited till the last follow‐up. Moreover, novel biomarkers including HPV and P16 status might be promising candidates, yet they were not routinely tested at our center. Finally, reproductivity of the findings should be checked in patients receiving other chemotherapy regimens.

## 5 CONCLUSION

Continuous high levels of SCC‐A indicated poorer tumor responses and long‐term outcomes of PC patients after TIP chemotherapy. SCC‐A might assist tumor monitoring of advanced PC after systemic therapies.

## AUTHOR CONTRIBUTIONS

**Nan Ma:** Conceptualization (equal); data curation (equal); formal analysis (equal); methodology (equal); writing – original draft (equal). **Yi‐Xiang Gan:** Conceptualization (equal); formal analysis (equal); methodology (equal). **Zhen‐Hua Liu:** Funding acquisition (equal); supervision (equal); writing – review and editing (equal). **Yin‐Yao Chao:** Data curation (equal); resources (equal). **Xian‐Da Chen:** Resources (equal); software (equal). **Kai Yao:** Resources (equal); supervision (equal); writing – review and editing (equal). **Hui Han:** Funding acquisition (equal); project administration (equal); resources (equal); supervision (equal). **Sheng‐Jie Guo:** Conceptualization (equal); resources (equal); supervision (equal); writing – review and editing (equal).

## FUNDING INFORMATION

Guangdong Province Nature Foundation of China Project (Grant Numbers: 2021A1515220182, 2022A1515012200, 2022A1515012321).

## CONFLICT OF INTEREST STATEMENT

The authors declare no conflicts of interest.

## ETHICS STATEMENT

This retrospective observational study has gained approval (approval number: B2023‐390‐01; approval date: August 16, 2023) from the ethics committee of the Sun Yat‐sen University Cancer Center.

## CONSENT

The informed consent requirement was waived due to the retrospective nature of the study and patient information was kept confidential.

## Data Availability

The data can be applied for via email contact with Sheng‐Jie Guo, the corresponding author.
